# Exploring bikeability in a metropolitan setting: stimulating and hindering factors in commuting route environments

**DOI:** 10.1186/1471-2458-12-168

**Published:** 2012-03-08

**Authors:** Lina Wahlgren, Peter Schantz

**Affiliations:** 1 The Research Unit for Movement, Health and Environment, The Åstrand Laboratory, GIH - The Swedish School of Sport and Health Sciences, SE-114 86 Stockholm, Sweden; 2 School of Health and Medical Sciences, Örebro University, SE-701 82 Örebro, Sweden; 3 Department of Health Sciences, Mid Sweden University, SE-831 25 Östersund, Sweden

## Abstract

**Background:**

Route environments may influence people's active commuting positively and thereby contribute to public health. Assessments of route environments are, however, needed in order to better understand the possible relationship between active commuting and the route environment. The aim of this study was, therefore, to assess the potential associations between perceptions of whether the route environment on the whole hinders or stimulates bicycle commuting and perceptions of environmental factors.

**Methods:**

The Active Commuting Route Environment Scale (ACRES) was used for the assessment of bicycle commuters' perceptions of their route environments in the inner urban parts of Greater Stockholm, Sweden. Bicycle commuters (n = 827) were recruited by advertisements in newspapers. Simultaneous multiple regression analyses were used to assess the relation between predictor variables (such as levels of exhaust fumes, noise, traffic speed, traffic congestion and greenery) and the outcome variable (hindering - stimulating route environments). Two models were run, (Model 1) without and (Model 2) with the item *traffic: unsafe or safe *included as a predictor.

**Results:**

Overall, about 40% of the variance of hindering - stimulating route environments was explained by the environmental predictors in our models (Model 1, *R^2 ^*= 0.415, and Model 2, *R ^2^*= 0.435). The regression equation for Model 1 was: y = 8.53 + 0.33 *ugly or beautiful *+ 0.14 *greenery *+ (-0.14) *course of the route *+ (-0.13) *exhaust fumes *+ (-0.09) *congestion: all types of vehicles *(*p *≤ 0.019). The regression equation for Model 2 was y = 6.55 + 0.31 *ugly or beautiful *+ 0.16 *traffic: unsafe or safe *+ (-0.13) *exhaust fumes *+ 0.12 *greenery *+ (-0.12) *course of the route *(*p *≤ 0.001).

**Conclusions:**

The main results indicate that beautiful, green and safe route environments seem to be, independently of each other, stimulating factors for bicycle commuting in inner urban areas. On the other hand, exhaust fumes, traffic congestion and low 'directness' of the route seem to be hindering factors. Furthermore, the overall results illustrate the complexity of a research area at the beginning of exploration.

## Background

In many countries, increasing the population's level of physical activity is a major public health concern e.g. [1]. Active commuting could constitute an important potential in this respect, not least since a lack of time appears to be a major hindrance to physically active behaviours cf. [2]. In a review on the theme 'Is active commuting the answer to population health?', Roy Shephard stated that more research concerning the impact of active commuting on population health is needed and that 'More objective information is also needed on how to persuade the general population to engage in active commuting; this should involve studies not only of counselling, but also of the built environment; how could simple and more complex modifications of the urban landscape encourage active transportation?' [3], p. 756. We agree, and the focus of this study is on the potential importance of the route environment for active commuting behaviours cf. [4].

Studies on the relationship between physical activity and the physical environment, as well as the traffic environment, are a relatively new research area. They hardly existed before the new millennium, but the field has expanded markedly during the past decade [5]. The predominant aim of this research area has been to try to understand how environments may affect levels of physical activity within the population. For this purpose, a number of research strategies have been developed. All of them have strengths and weaknesses.

Giles-Corti and colleagues [6] indicated a principal problem in this research area, namely a lack of specificity concerning both the type and purpose of the physical activity and the environment within which the behaviour occurs. This lack of specificity can still be noted. Physical activity can be specified by type, such as walking or cycling, and it can be done for a specific purpose, such as exercise or leisure. Physical activity carried out with the purpose of active transport can be specified by the purpose or the destination of the trip, such as bicycle commuting to one's place of work. Thus, bicycle commuting to one's place of work is a specific physical activity, and the associated route environment is the specific physical activity environment.

To fully understand the effect of environment on physical activity behaviours, it would be preferable to be able to isolate the effect of different environmental variables *per se*. This demands controlling for variations in all other variables of importance, and it is a difficult task. However, in line with this thinking, there is a need to differentiate between and within potential environmental categories of importance.

We have recently elaborated on this, framed it within the term *bikeability*, and concluded that whether the route environment is perceived as stimulating or hindering active commuting is an integrative environmental category of potential importance [7]. It may affect the behaviour related to bicycling, as well as the degree, or lack, of well-being of bicyclists when cycling. Thus, studying bicycle commuters' perceptions of their route environment constitutes an important research area.

The Active Commuting Route Environment Scale (ACRES) has been developed for the assessment of bicyclists' perceptions of their self-chosen commuting route environment [8]. It is based on a complete spatial matching of the environment and the physical activity variable, and has shown considerable criterion-related validity and reasonable test-retest reproducibility [7,8].

In two previous studies we have used the ACRES to assess a metropolitan setting [7,8], and noted that bicycle commuters generally rated suburban route environments as more stimulating for bicycle commuting than inner urban ones. At the same time, for example, higher ratings of greenery and lower ratings of exhaust fumes, noise and the flow of motor vehicles were noted in the suburban than in the inner urban route environments. These environmental factors were therefore regarded as potential explanatory factors in relation to what constitutes the overall perception of whether a route environment is hindering or stimulating for bicycle commuting.

The aim of this study is to further assess these potential associations, but here we make use of the ACRES together with another analytical approach, namely multiple regression analyses. For this purpose, adult active commuters were recruited in Greater Stockholm, Sweden. Data on these bicycle commuters' perceptions of their individual route environments in the inner urban area of Stockholm were used. Since Stockholm has a variety of settings with distinctly different environmental characteristics, this approach was expected to result in relatively large individual variations of ratings, and thereby enable an exploratory comparative study of the relations between different items.

## Methods

### Participants and procedure

The participants were recruited with the aim of attaining a reasonable representation of the adult active commuters in the inner urban and suburban areas of Greater Stockholm during the recruitment period. Active commuters constitute a small group within the general population and therefore it was not possible, in practical terms, to recruit a sufficient number of participants from a random population sample. We therefore recruited participants by advertising in two large morning newspapers in Stockholm (Dagens Nyheter and Svenska Dagbladet) towards the end of May and early June 2004. Inclusion criteria were: (a) being at least 20 years old; (b) living in Stockholm County, excluding the municipality of Norrtälje; and (c) walking and/or cycling the whole way to one's place of work or study at least once a year. In the invitation to participate, we emphasized that people with short commuting distances were also welcome to participate. The reason for including people with less frequent active commuting behaviours, as well as with short route distances, was to include a wide range of commuting behaviours.

The advertisements resulted in 2,148 individuals volunteering to take part. We posted a first questionnaire, called the Physically Active Commuting in Greater Stockholm Questionnaire (PACS Q1; for further description, see below), to the participants in September 2004. The response frequency was 94% (n = 2010). During the peak bicycle-commuting period of the year, in May 2005, a second questionnaire, the PACS Q2, was sent to 1978 participants. The response frequency was 92% (n = 1819). Both questionnaires were sent home to each participant together with a prepaid return envelope. A maximum of three reminders were sent out. No incentives were provided for participation. We excluded some participants in the second round because they did not meet the inclusion criteria, or did not wish to participate in the second part of the study. The participants were bicyclists, pedestrians or dual-mode commuters, i.e. individuals who sometimes walk and sometimes cycle. They commuted in the inner urban or suburban - rural areas of Greater Stockholm, or both of these areas. Since these areas represent distinctly different environmental settings (for details, see [7]), we believe that it is of clear importance, at least initially, to study them as separate entities. In this study we have therefore only used data on bicycle commuting in the inner urban area. After cleansing and editing the data, 827 participants (women, n = 499, 60%) were included in the analyses. We were concerned about whether or not bicycling and ratings of route environments in two different areas, as compared to only one area, would affect the rating levels. Therefore, in a previous study [7], partly based on the same participants as those in this study, we compared ratings of inner urban route environment between those who bicycle commuted in both inner urban and suburban - rural areas (n = 555) and those who bicycle commuted in only an inner urban area (n = 272). Overall, the results indicated only few and small differences between the groups. Hence, in this study we combined the two groups. For further descriptive characteristics of the participants, see Table 1.

**Table 1:** Descriptive characteristics of participants (n = 816-826)

Characteristic	
Females, %	60
Age in years, mean ± SD	47.4 ± 10.7
Weight in kg, mean ± SD	69.9 ± 11.4
Height in cm, mean ± SD	173.4 ± 8.7
Body mass index, mean ± SD	23.2 ± 2.8
Gainful employment, %	94
Educated at university level, %	78
An income above 25 000 SEK* a month, %	65
Participant and both parents born in Sweden, %	82
Having a driver's licence, %	94
Usually access to a car, %	71
Leaving home 7-9 a.m. to cycle to work, %	75
Number of bicycle-commuting trips per year**, mean ± SD	279 ± 133
Overall physical health either good or very good, %	84
Overall mental health either good or very good, %	84

Values are based on self-reports

*SEK Swedish crown/krona, year 2005: €1 ≈ 9 SEK; US$1 ≈ 8 SEK

**The number of bicycle-commuting trips per year is based on 681 participants. The low response rate is due to missing values in one or more of the 12 months leading to exclusion in the sum score

We were also concerned about the representativity of the advertisement-recruited participants. We therefore, in a previous study [7], compared the ratings of route environments between advertisement- and street-recruited participants. The street recruitment strategy was considered to represent the population of active commuters at that period of recruitment with greater certainty than the advertisement strategy. Overall, the results indicated a good correspondence between the advertisement- and street-recruited participants' ratings. For example, the sex-neutral mean values for the different items for the different groups were gathered along the line of identity for both urban and suburban areas, and the Pearson correlation coefficients were 0.96-0.98 [7].

The Ethics Committee of the Karolinska Institute approved the study. The participants gave their informed consent.

### The physically active commuting in Greater Stockholm questionnaire (PACS Q)

The PACS Q1 and PACS Q2 are self-administered questionnaires in Swedish, based on self-reports. They include 35 and 68 items, respectively, comprising descriptive characteristics of participants and different aspects of active commuting. The PACS Q2 includes the ACRES.

### Measure of descriptive characteristics

Data on sex, age, weight, height, employment and number of bicycle-commuting trips per month were obtained from the PACS Q1. The body mass index (BMI) was calculated by dividing body weight by height squared (kg·m^-2^). Active commuting trips per year were calculated by adding each month's average trip frequency per week and then dividing the sum by 12 to obtain values for an 'average week', which were thereafter multiplied by 52. Education levels, income, ethnicity, having a driver's licence, having access to a car, time leaving home to cycle to work and overall physical and mental health were obtained from the PACS Q2 (Table 1).

### The active commuting route environment scale (ACRES)

The ACRES consists of 18 items for the assessment of bicyclists' perceptions of their self-chosen commuting route environment, potentially associated with active commuting. A more detailed description of the development of the ACRES, its items and its validity and reliability has been reported elsewhere [7,8]. The ACRES was characterized by considerable criterion-related validity and reasonable test-retest reproducibility.

**Figure 1:**
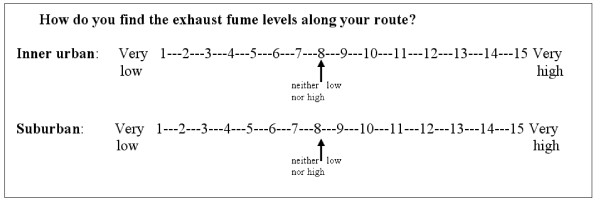
**Example of an item from the Active Commuting Route Environment Scale (ACRES) for bicyclists**. The participants were asked to differentiate between their experiences when their active commuting route is in the inner urban area and when it is in the surrounding suburban as well as rural areas (see Figure 2 for the distinction between these areas). If the participants cycle in both areas, they are asked to mark both lines. If the participants, for instance, first cycle in the southern suburban area, then cross into the inner urban area and finish their route in the northern suburban area, then they are asked to give an average rating for both suburban areas of the route. The same refers to cases where the participants make use of different inner urban route environments that are interspersed with suburban - rural ones.

Each item considers the inner urban area of Stockholm, the capital of Sweden, and the suburban as well as rural areas surrounding it, within Stockholm County, separately. The questionnaire instructions include a drawn map that distinguishes the inner urban area from the surrounding areas, see [8]. The participants were asked to differentiate between their experiences when their active commuting route is in the inner urban area and when it is in the surrounding suburban as well as rural areas (Figure 1). All items have two identical parallel response lines. One line refers to the inner urban area and the other to the suburban as well as rural areas. The separation between these parts was based essentially on the fact that they constitute different environments: the inner urban area is a dense urban setting with blocks placed in a grid-like streetscape, typical of European cities, whereas, with very few and small exceptions, this is not the case in the suburban-rural areas. For a detailed description of each area's environmental features, see [7].

To simplify understanding, the items for the assessment of bicyclists' perceptions have been divided into: (a) the physical environment; (b) the traffic environment; and (c) the social environment. The following items are included in the physical environment (see Table 2): *bicycle paths/lanes/roads *(#11), *greenery *(#13), *ugly or beautiful *(#14), *course of the route *(#15), *hilliness *(#16), *red lights *(#17) and *short or long *(#18). They represent non-moving aspects. The following items are included in the traffic environment: *exhaust fumes *(#3), *noise *(#4), *flow of motor vehicles *(#5), *speeds of motor vehicles *(#6), *speeds of bicyclists *(#7), *congestion: all types of vehicles *(#8) and *congestion: bicyclists *(#9). They represent moving aspects. The following item is included in the social environment: *conflicts *(#10). It represents relationships between road users. All items are meant to operate independently. The remaining three items, namely, *on the whole *(#1), *hinders or stimulates *(#2) and *traffic: unsafe or safe *(#12), are regarded as outcome variables. All the other items are regarded as predictor variables believed to be potentially important for the outcome variables. The numbers specified in parentheses indicate the order in the questionnaire; see Table 2. In this study *short or long *and *on the whole *are not used.

**Table 2:** The Active Commuting Route Environment Scale (ACRES) for bicyclists

	15-point response scale	
	
Question	1	15
1. How do you experience the environment on the whole along the route?	Very bad	Very good
2. Do you think that, on the whole, the environment you cycle in stimulates/hinders your commuting?	Hinders a lot	Stimulates a lot
3. How do you find the exhaust fume levels along your route?	Very low	Very high
4. How do you find the noise levels along your route?	Very low	Very high
5. How do you find the flow of motor vehicles (number of cars) along your route?	Very low	Very high
6. How do you find the speeds of motor vehicles (taxis, lorries, ordinary cars, buses) along your route?	Very low	Very high
7. How do you find other cyclists' speeds along your route?	Very low	Very high
8. How do you as a cyclist find the congestion levels in mixed traffic, caused by all types of vehicles, along your route?	Very low	Very high
9. How do you find the congestion levels caused by the number of cyclists on the cycle paths/cycle lanes along your route?	Very low	Very high
10. How do you find the occurrence of conflicts between you as a cyclist and other road users (including pedestrians) along your route?	Very low	Very high
11. About how large a part of your route consists of cycle paths/cycle lanes/cycle roads separated from motor-car traffic?	0%	100%*
12. How unsafe/safe do you feel in traffic as a cyclist along your route?	Very unsafe	Very safe
13. How do you find the availability of greenery (natural areas, parks, planted items, trees) along your route?	Very low	Very high
14. How ugly/beautiful do you find the surroundings along your route?	Very ugly	Very beautiful
15. To what extent do you feel that your cycle trip is made more difficult by the course of the route? For example, a course with many sharp turns, detours, changes in direction, side changeovers etc.	Very little	Very much
16. To what extent do you feel that your cycle trip is made more difficult by hilliness? Base this on the route to and from your place of work/study.	Very little	Very much
17. To what extent do you feel that your progress in traffic is worsened by the number of red lights during your trip to your place of work/study?	Very little	Very much
18. How short/long do you experience your route to be?	Very short	Very long

Note that this is a translation of the original ACRES in Swedish

*11-point scale

The reason for not using the perception of *short and long *is that it refers to the whole trip distance and the fact that a significant portion of our participants do also cycle in the suburban area. The reason for not using the perception of *on the whole *is that this item is too general for the purpose of this study. On the other hand, given that this is an exploratory analysis of what constitutes the overall perception of whether a route environment is hindering or stimulating, we have included *traffic: unsafe or safe *(which normally is viewed as an outcome variable) as a predictor variable in model 2 in our analysis (see below). This is to check if there are indications that there might be an overlapping environmental basis for these two different outcome variables.

Fifteen-point response scales, with adjectival opposites, ranging from 1 to 15, corresponding to, for example, 'very low' and 'very high', are used, with the exception of one item. The item *bicycle paths/lanes/roads *has an 11-point response scale ranging from 0% (0) to 100% (10) (Table 2). The 15-point response scales feature a numbered continuous line, i.e. whole numbers from 1 to 15, with number 8 as a neutral option in the middle, labelled, for example, 'neither low nor high' (Figure 1).

In the questionnaire instructions, the participants are asked to recall and rate their overall experience of their self-chosen route environments based on their active commuting to their place of work or study during the previous 2 weeks. The reason for this is that we wanted them to have fresh perceptions. Individuals stating that they had not been cycling the last 2 weeks were therefore excluded. At no point are the participants informed about the intent of the ACRES.

### Study area

**Figure 2:**
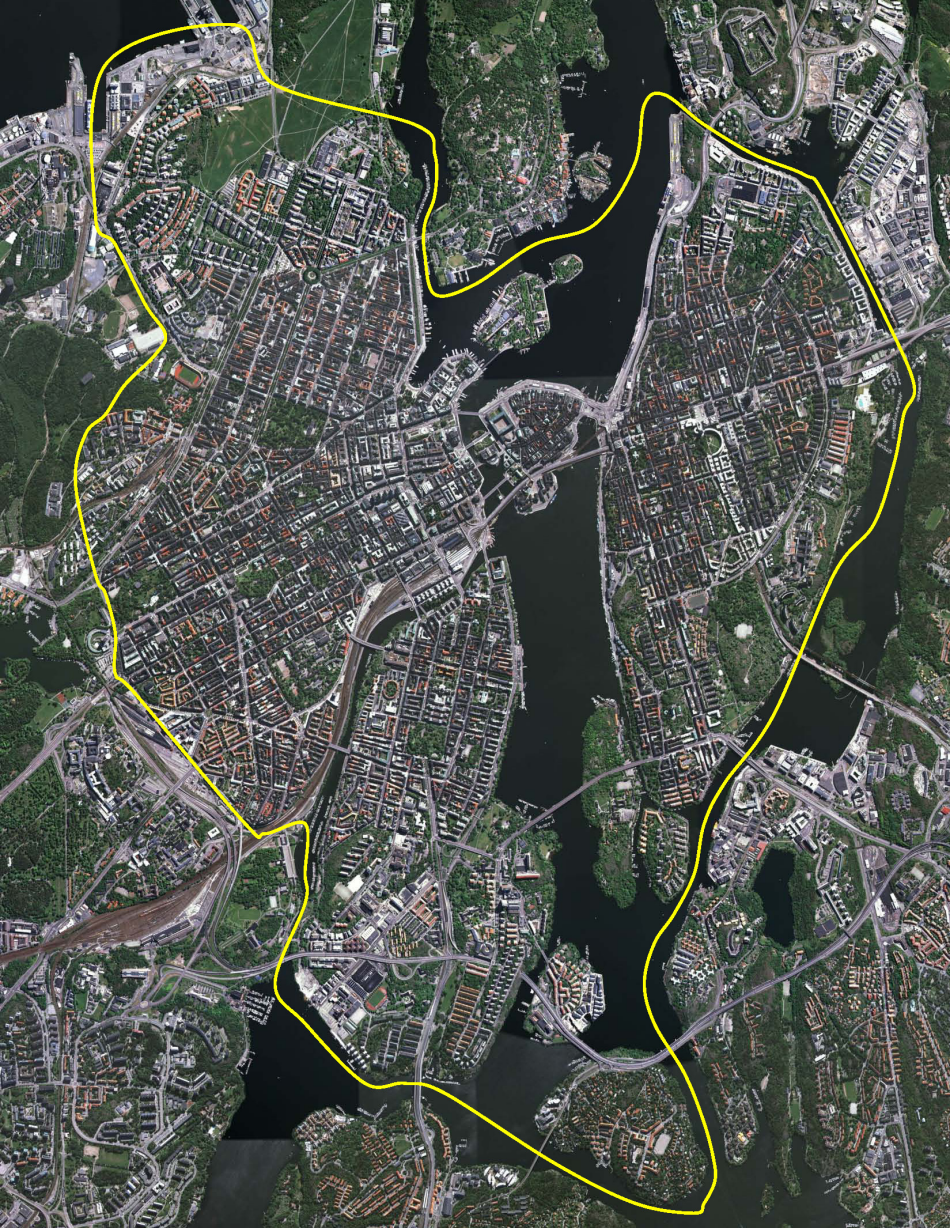
**Aerial view from 2005 over the inner urban parts of Greater Stockholm, Sweden**. The yellow line distinguishes the inner urban and suburban - rural parts. North is on the left of the image. For description of the characteristics of the study area, see Methods. (Copyright: Lantmäteriverket, Gävle, Sweden, 2011; Permission 81055230.).

The commuting route environments are located in the inner urban area of Stockholm, the capital of Sweden, in the centre of a metropolitan area with about 1.9 million inhabitants. This area constitutes the region's single core urban structure, with the centre situated where Lake Mälaren meets the Baltic Sea, thereby dividing the region into two main parts. The study area includes the city sections of 'Gamla stan' (the Old Town), Södermalm, Kungsholmen, Vasastan, Norrmalm and Östermalm (Figure 2). This is a predominantly built-up area, with blocks in a grid-like streetscape. The age of the buildings varies. The Old Town is from medieval times, whereas most parts of the built-up environment are predominantly a result of the architectural styles from the end of the 19th and beginning of the 20th century, with most buildings about five storeys high. The newest part of the city centre is north of the Old Town. The original buildings here were torn down during the 1950s and 1960s, and today the area includes modernistic architecture, including a few skyscrapers. In 2005 the residential density of the inner urban parts of the study area was approximately 13 000 residents per square km [9].

The city has a number of waterfronts and islands, a number of both small and large parks, some alleys and esplanades. Most streets are void of trees or other forms of greenery. The natural landscape in the area is sediment-filled valleys as a part of the surrounding rift-valley landscape and raised archipelago landscape with eroded bedrocks after deglaciation. It is basically rather flat, but there are some dominant natural features such as, for example, part of an esker, rising 40 m above sea level in Vasastan, as well as a rather steep fault scarp in Södermalm. The road system also includes rather gentle slopes of infrequent moraine hills, normally not accounting for more than about 10-15 m of elevation. Two arterial highways pass through the inner urban area (Centralleden and Essingeleden), but cyclists or pedestrians come into very little contact with them. These are also the only roads, besides some tunnels, that do not permit cycling.

### Statistical analyses

Questionnaire data were entered in the Statistical Package for the Social Sciences, version 19.0 (IBM SPSS Inc., Somer, NY, USA). All entered data from the PACS Q2 were checked for accuracy. Some participants were excluded, mainly because of incorrect or incomplete ACRES data. Participants with three or less missing ACRES values for cyclists were used for the following measures: (1) percentages and mean scores ± 1 standard deviation (SD), used to report the characteristics of the participants.; (2) the values of the ACRES items, presented as mean scores ± 1 SD; and (3) interrelations between the variables assessed with Pearson's correlation coefficient (*r*).

Simultaneous multiple regression analysis was chosen to explore associations between the outcome variable, *hinders or stimulates*, and the predictor variables *exhaust fumes*, *noise*, *flow of motor vehicles*, *speeds of motor vehicles*, *speeds of bicyclists*, *congestion: all types of vehicles*, *congestion: bicyclists*, *conflicts*, *bicycle paths/lanes/roads*, *traffic: unsafe or safe*, *greenery*, *ugly or beautiful*, *course of the route*, *hilliness *and *red lights*. Two models were run. In Model 1, *traffic: unsafe or safe *was excluded, and in Model 2, it was included as a predictor. The reason for including *traffic: unsafe or safe*; a variable that we normally regard as an outcome variable, was, as stated previously, its possible association with the outcome variable: *hinders or stimulates*. Only participants that had no missing values for any of the studied ACRES variables were used in the simultaneous multiple regression analyses.

Before running the simultaneous multiple regression analyses, linearity of the variables was assessed visually by means of scatterplots, boxplots and errorbars. All variables demonstrated reasonable linearity and were therefore used in the analyses. Furthermore, before the analyses, interrelations between the variables were assessed with Pearson's correlation coefficient (see Table 3). The correlations between predictor variables were, in absolute values, *r *≤ 0.68, indicating no problems with multicollinearity. In addition, multicollinearity was checked with the variance inflation factor (VIF). Both models' VIFs (all values ≤ 2.26, mean: 1.65) indicated no problem with multicollinearity.

**Table 3:** Correlations between ratings of environmental variables (n = 818-827)

Variable	1	2	3	4	5	6	7	8	9	10	11	12	13	14	15	16
1. Hinders or stimulates	-															
2. Exhaust fumes	-0.35*	-														
3. Noise	-0.30*	0.68*	-													
4. Flow of motor vehicles	-0.32*	0.58*	0.60*	-												
5. Speeds of motor vehicles	-0.22*	0.35*	0.43*	0.49*	-											
6. Speeds of bicyclists	0.01	0.20*	0.25*	0.23*	0.39*	-										
7. Congestion: all types of vehicles	-0.32*	0.41*	0.40*	0.50*	0.39*	0.22*	-									
8. Congestion: bicyclists	-0.08*	0.22*	0.22*	0.29*	0.24*	0.39*	0.50*	-								
9. Conflicts	-0.21*	0.23*	0.19*	0.26*	0.18*	0.09*	0.45*	0.45*	-							
10. Bicycle paths/lanes/roads	0.21*	-0.05	0.03	-0.03	-0.05	0.14*	-0.15*	0.14*	-0.03	-						
11. Traffic: unsafe or safe	0.44*	-0.28*	-0.28*	-0.31*	-0.32*	-0.09*	-0.47*	-0.24*	-0.34*	0.26*	-					
12. Greenery	0.48*	-0.32*	-0.28*	-0.30*	-0.18*	0.06	-0.31*	-0.07*	-0.14*	0.26*	0.37*	-				
13. Ugly or beautiful	0.52*	-0.21*	-0.23*	-0.18*	-0.11*	0.11*	-0.12*	0.07	-0.06	0.23*	0.28*	0.54*	-			
14. Course of the route	-0.31*	0.12*	0.12*	0.15*	0.12*	-0.08*	0.24*	0.17*	0.30*	-0.15*	-0.34*	-0.17*	-0.18*	-		
15. Hilliness	0.00	0.01	0.05	0.05	0.05	0.13*	0.09*	0.14*	0.14*	0.05	-0.01	0.03	0.04	0.19*	-	
16. Red lights	-0.30*	0.28*	0.29*	0.36*	0.21*	0.00	0.41*	0.19*	0.32*	-0.13*	-0.31*	-0.32*	-0.18*	0.35*	0.13*	-

*p ≤ 0.05

The top limit for inclusion of standardized residuals in the models was set to ± 4 SD, according to the sample size used [10]. Possible extreme data cases were defined using Cook's distance. No extreme data cases could be defined using Cook's distance in either of the models (all values ≤ 0.03, mean: 0.001).

Sex (dichotomous categorical variable), age (continuous variable), education (categorical variable coded as dichotomous: university/university college or other lower) and income (categorical variable coded as three categories and used as a dummy variable: ≤ 25 000 SEK, 25 001-30 000 SEK or ≥ 30 001 SEK; SEK = Swedish crown/krona, year 2005: €1 ≈ 9 SEK; US$1 ≈ 8 SEK) were possible confounding variables. Before considering using them in the simultaneous multiple regression analyses, we assessed their individual contribution to the variation in the outcome variable using simple regression analyses for the sex, age and education variables, and simultaneous multiple regression analysis for the income variable. The results demonstrated no significant contribution or a very small significant contribution (age: *R^2 ^*= 0.008). We have therefore chosen not to include these variables in the simultaneous multiple regression analyses.

The values from the simultaneous multiple regression analyses are presented as unstandardized beta coefficients (*B*) and their 95% confidence interval (CI), and partial correlation coefficients. Furthermore, the R square (*R^2^*) is presented for the overall models.

A statistical level corresponding to at least *p *≤ 0.05 was used to indicate significance.

## Results

Interrelations between all variables and their mean scores are shown in Tables 3 and 4, respectively. The range for correlations between the outcome variable hinders or stimulate and the predictor variables was, in absolute values, *r *= 0.00 - 0.52. The following items had a positive correlation (*p *≤ 0.05) with the outcome variable: *ugly or beautiful *(*r *= 0.52), *greenery *(*r *= 0.48), *traffic: unsafe or safe *(*r *= 0.44), and *bicycle paths/lanes/roads *(*r *= 0.21). The following items had a negative correlation (p ≤ 0.05) with the outcome variable: *congestion: bicyclists *(*r *= -0.08), *conflicts *(*r *= -0.21), *speeds of motor vehicles *(*r *= -0.22), *noise *(*r *= -0.30), *red lights *(*r *= -0.30), *course of the route *(*r *= -0.31), *flow of motor vehicles *(*r *= -0.32), *congestion: all types of vehicles *(*r *= -0.32) and *exhaust fumes *(*r *= -0.35). *Speeds of bicyclists *was close to no significant correlation with the outcome variable (*r *= 0.01) and *hilliness *had no correlation with the outcome variable (*r *= 0.00).

**Table 4:** Participants' ratings of environmental variables (n = 821-827)

Variable	Mean ± SD	15-point response scale	
		1	15
Hinders or stimulates	9.16 ± 3.32	Hinders a lot	Stimulates a lot
Exhaust fumes	9.91 ± 3.15	Very low	Very high
Noise	9.62 ± 3.04	Very low	Very high
Flow of motor vehicles	11.14 ± 3.34	Very low	Very high
Speeds of motor vehicles	9.45 ± 2.83	Very low	Very high
Speeds of bicyclists	9.17 ± 2.85	Very low	Very high
Congestion: all types of vehicles	10.45 ± 3.30	Very low	Very high
Congestion: bicyclists	8.93 ± 3.73	Very low	Very high
Conflicts	8.27 ± 3.73	Very low	Very high
Bicycle paths/lanes/roads	5.86 ± 2.87	0%	100%*
Traffic: unsafe or safe	8.53 ± 3.69	Very unsafe	Very safe
Greenery	7.08 ± 4.02	Very low	Very high
Ugly or beautiful	10.12 ± 3.28	Very ugly	Very beautiful
Course of the route	6.99 ± 3.83	Very little	Very much
Hilliness	5.10 ± 3.54	Very little	Very much
Red lights	8.18 ± 4.31	Very little	Very much

*Percentage values have been transformed into an 11-point scale. Minimal value = 0 and maximal value = 10. For the questions associated with the variables, see Table 2

The results of the analysis for Model 1 (in which the item *traffic: unsafe or safe *was excluded) are shown in Table 5. About 40% of the variance of the outcome variable, *hinders or stimulate*, was explained by the environmental predictors in the model (*R^2 ^*= 0.415). The regression equation was: y = 8.53 + 0.33 *ugly or beautiful *+ 0.14 *greenery *+ (-0.14) *course of the route *+ (-0.13) *exhaust fumes *+ (-0.09) *congestion: all types of vehicles *(*p *≤ 0.019).

**Table 5:** Simultaneous multiple regression analysis of route environment variables (Model 1, excluding *traffic: unsafe or safe*) (n = 805)

Outcome variable	y-intercept	p-value	95% CI	
**Hinders or stimulates**	**8.53**	**0.000**	**7.34 - 9.72**	

	**Regression coefficient**			**Partial correlation** **coefficient**
		
**Predictor variable**	***B***	**p-value**	**95% CI**	

Exhaust fumes	-0.13	0.002	-0.21 - -0.05	-0.11
Noise	0.01	0.800	-0.08 - 0.10	0.01
Flow of motor vehicles	-0.04	0.309	-0.12 - 0.04	-0.04
Speeds of motor vehicles	-0.04	0.368	-0.11 - 0.04	-0.03
Speeds of bicyclists	0.00	0.905	-0.08 - 0.07	0.00
Congestion: all types of vehicles	-0.09	0.019	-0.17 - -0.02	-0.08
Congestion: bicyclists	0.05	0.146	-0.02 - 0.11	0.05
Conflicts	-0.05	0.110	-0.11 - 0.01	-0.06
Bicycle paths/lanes/roads*	0.03	0.407	-0.04 - 0.10	0.03
Traffic: unsafe or safe	-	-	-	-
Greenery	0.14	0.000	0.09 - 0.20	0.18
Ugly or beautiful	0.33	0.000	0.27 - 0.40	0.33
Course of the route	-0.14	0.000	-0.20 - -0.09	-0.19
Hilliness	0.03	0.205	-0.02 - 0.09	0.05
Red lights	-0.02	0.394	-0.07 - 0.03	-0.03

*R^2 ^*= 0.415

*Minimal value = 0 and maximal value = 10

The results of the analysis for Model 2 (in which the item *traffic: unsafe or safe *was included as a predictor) are shown in Table 6. About 40% of the variance of the outcome variable *hinders or stimulate *was explained by the environmental predictors in the model (*R^2 ^*= 0.435). The regression equation was: y = 6.55 + 0.31 *ugly or beautiful *+ 0.16 *traffic: unsafe or safe *+ (-0.13) *exhaust fumes *+ 0.12 *greenery *+ (-0.12) *course of the route *(*p *≤ 0.001).

**Table 6:** Simultaneous multiple regression analysis of route environment variables (Model 2, including *traffic: unsafe or safe*) (n = 805)

Outcome variable	y-intercept	p-value	95% CI	
**Hinders or stimulates**	**6.55**	**0.000**	**5.17 - 7.93**	

	**Regression coefficient**			**Partial correlation** **coefficient**
		
**Predictor variable**	***B***	**p-value**	**95% CI**	

Exhaust fumes	-0.13	0.001	-0.21 - -0.05	-0.11
Noise	0.01	0.733	-0.07 - 0.10	0.01
Flow of motor vehicles	-0.05	0.240	-0.12 - 0.03	-0.04
Speeds of motor vehicles	-0.01	0.857	-0.08 - 0.07	-0.01
Speeds of bicyclists	0.00	0.999	-0.07 - 0.07	0.00
Congestion: all types of vehicles	-0.05	0.216	-0.12 - 0.03	-0.04
Congestion: bicyclists	0.05	0.130	-0.01 - 0.11	0.05
Conflicts	-0.03	0.380	-0.08 - 0.03	-0.03
Bicycle paths/lanes/roads*	0.00	0.908	-0.06 - 0.07	0.00
Traffic: unsafe or safe	0.16	0.000	0.10 - 0.22	0.19
Greenery	0.12	0.000	0.07 - 0.18	0.15
Ugly or beautiful	0.31	0.000	0.25 - 0.38	0.32
Course of the route	-0.12	0.000	-0.17 - -0.06	-0.15
Hilliness	0.02	0.402	-0.03 - 0.07	0.03
Red lights	-0.02	0.458	-0.07 - 0.03	-0.03

*R^2 ^*= 0.435

*Minimal value = 0 and maximal value = 10

## Discussion

This is, to our knowledge, one of the first exploratory studies on bicyclists' perceptions of their self-chosen commuting route environment, based on a complete spatial matching of the environment and the physical activity variable. The overall results demonstrate a complex research area at the beginning of exploration. The main results indicate that in inner urban areas, the factors beautiful, green and safe route environments seem to, independently of one another, be stimulating for bicycle commuting. On the other hand, exhaust fumes, traffic congestion, and low 'directness' of the route, seem to be hindrances to bicycle commuting.

As mentioned in the Background, we have previously used the ACRES to compare inner urban and suburban route environments in the metropolitan setting of Greater Stockholm [7,8]. In general, bicycle commuters rated suburban route environments as more stimulating for bicycle commuting than inner urban route environments. At the same time, for example, higher ratings of greenery and lower ratings of exhaust fumes, traffic congestion and directness of the route were noted in the suburban as compared to the inner urban route environments. These factors were therefore regarded as potential explanatory factors in relation to hindering - stimulating route environments. Thus, we now have different types of evidence supporting this role of these factors. Interestingly, in the present study, beauty, which did not differ significantly between inner urban and suburban route environments [7], stands out as an additional factor of importance for stimulating bicycle commuting.

### A broader perspective

Before discussing our findings in more detail, we will place our study in a broader perspective. It is based on a selected group. We believe that this is necessary in order to acquire this kind of knowledge, since in this case the bicycle commuters are the experts themselves. The value of using this more uniform group is also the minimization of the effect of confounders. For example, our results cannot be due to self-selection factors as a result of a cross-sectional study design. Furthermore, our results are not likely to be influenced by the risk that people state what they are expected to state due to, for example, what is proposed in policy documents. Otherwise, this is a possible risk in surveys. The reason for this judgement in relation to the ACRES is that its aim is never presented to the participants, and that the order of items in the ACRES has the outcome variable *hinders or stimulate *before the predictor items (cf. Table 2). Thus, ratings of the outcome variable are probably not affected by an awareness of different predictors. On the other hand, using a selected group prompts the question about external validity. We will comment further on these types of issues later in the Discussion.

Additionally, this study includes both potentially stimulating and hindering environmental variables in the analyses. Therefore, we can control for their independent effects. For example, in our study, *greenery *correlates negatively with *exhaust fumes*, *noise *and *flow of motor vehicles*. It is therefore impossible to state anything about the association per se of *greenery*, without simultaneously controlling for the associations of the other mentioned variables. However, the design of our study allows for this.

### The models

Given this broader perspective, a more detailed discussion of the results will follow here. Overall, in our models, about 40% of the variance of the outcome variable *hinders or stimulate*, was explained by the environmental predictors. Some of the unexplained variance can be due to the level of reproducibility of the scale [8] or that factors of importance might be missing. Variables of possible importance are real distance, perceived distance, and if the distance is perceived as short or long. Another variable of possible importance is perceived exertion. These factors might possibly affect the perceptions of both environmental predictors and outcome variables.

### Aesthetics and natural environments

**Figure 3:**
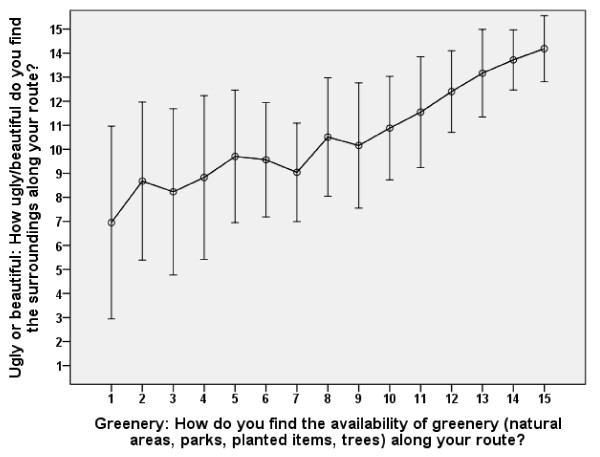
**The relationship between ratings of the route environmental variables *ugly or beautiful *and *greenery *in the inner urban area**. Mean ± 1 SD. N = 822. The Pearson's correlation coefficient is 0.54.

*Ugly or beautiful *was the predictor that contributed the most to the variance of the outcome variable in our models. It is, however, somewhat hard to interpret this finding. *Ugly or beautiful *is most likely a composite variable. In our study *greenery *was assessed as a separate factor. *Greenery *could, however, also be regarded as a part of *ugly or beautiful*. Correlation evidence in our study supports this relationship (see Table 3 and Figure 3). Yet, *greenery *and also *ugly or beautiful *were both factors that contributed positively to the variance of the outcome variable in our models. We interpret this in terms of that other forms of aesthetic features, such as architecture, water and open space, constitute an independent stimulating environmental impact on bicycle commuting.

Previous findings regarding aesthetics are somewhat contradictory. Pikora and colleagues [11] emphasized aesthetics as one of the features in a conceptual framework of environmental factors that may influence bicycling with different purposes. Aesthetics appears in their framework as a factor of importance for both recreational and transport bicycling. However, recreational bicycling included seven items of importance ('garden maintenance', 'street maintenance', 'cleanliness', 'pollution', 'parks', 'sights' and 'architecture'), whereas transport bicycling included only one item ('pollution'). This indicates that aesthetics was less important for transport bicycling. In line with this, perceptions of the 'aesthetic nature of the environment' have been found to be associated with walking for exercise or recreation, but not with walking for transport for a review, see [12]. The value of our findings in this respect is that they clearly point to the importance of aesthetics for transport bicycling.

Studies of bicycling in general terms, i.e. without a breakdown into different purposes, have indicated that the 'general attractiveness of the route' is a likely important factor cf. [13] and that 'routes with beautiful scenery' was regarded as a top motivator for current and potential bicyclists [14]. In contrast, Gebel and colleagues [15] found less-consistent associations between physical activity in general and 'aesthetic features' in a review of reviews. In general, however, aesthetics seem to have some positive association with physical activity in general for reviews, see [2,16,17]. Overall, and in combination with our own findings, these studies point toward a positive relation between aesthetics and physical activity in general. However, at the same time, the term aesthetics is used rather differently in different studies. Given the importance of this variable, as indicated in the present study, there appears to be a great need to sort out the important aspects of perceived aesthetics in different contexts of physical activity.

Although *greenery *could be regarded as a part of *ugly or beautiful*, it was, as mentioned, assessed as a separate factor and contributed positively to the variance of the outcome variable in our models. This may possibly be due to the fact that natural elements appear to be a positive modifier of stress and mood states cf. [18,19]. It is also possible that green elements represent fascinating external cues which compete with internal cues during physical exercise, leading to reduced levels of perceived exertion for a given intensity of physical activity [20].

Research on the relation between natural environments and bicycling is sparse and inconclusive. Nevertheless, Maas and colleagues [21] found a negative relation between the percentage of green space in a 1-km radius around people's homes and whether or not people bicycle-commuted. Indeed, a stated possibility was that in greener living environments, destinations, such as shops or places of work, tend to be further away, making distances less suitable for bicycling. If people bicycle-commuted, however, they were likely to spend more time on it if they had more green space around their homes. In accordance with the latter, Wendel-Vos and colleagues [22] found a positive relation between time spent on bicycle commuting and the amount of parks in the neighbourhood within a 300-m radius. In contrast, Moudon and colleagues [23] found no relation between the likelihood of bicycling in general in the neighbourhood and presence of parks in a home-based 3-km buffer area comprising peoples' residential environments. In addition, Winters and colleagues [24] studied the reasons for taking detours and did not find differences between the shortest and the actual routes depending on greenness: 'the percentage of land area with green cover, defined as street trees, park, park/forest trees, and grasslands'. One stated possible explanation was a lack of variability between the shortest route and detour within a reasonable distance.

These studies indicate the complexity and difficulties in studying these issues, and emphasize the importance of including distances as a key factor for cycling, particularly for understanding the behaviour of bicycle commuting. Irrespectively of having supporting route environments or not, bicycling will not take place if distances are viewed as not sufficiently short to undertake by bike. In contrast to these somewhat conflicting results, our findings clearly support a positive influence of greenery on bicycle commuting. Thus, other factors might influence the decision to cycle stronger than the presence of greenery, but once the cyclist is cycling in the route environment, greenery seems to be a stimulating factor.

### Road users and safety concerns

Bicycle commuting often involves interaction with other road users, such as motor vehicle drivers, pedestrians and other bicyclists. Two of our items that regard other bicyclists, *speeds of bicyclists *and *congestion: bicyclists*, both demonstrated low correlations with the outcome variable. Thus, other bicyclists do not appear to be a major hinder for the studied bicycle commuters. In contrast, the items regarding or associated with motor vehicles, *flow of motor vehicles*, *speeds of motor vehicles*, *congestion: all types of vehicles*, and *conflicts*, all demonstrated negative correlations with the outcome variable. In addition, *congestion: all types of vehicles *was one of the predictors that contributed negatively to the variance of the outcome variable in one of our models (Model 1, excluding *traffic: unsafe or safe*). Furthermore, two of our items associated with motor vehicles, i.e. *exhaust fumes *and *noise*, both demonstrated negative correlations with the outcome variable. *Exhaust fumes *was, in addition, one of the predictors that also contributed negatively to the variance of the outcome variable in our models. Thus, different aspects of motor vehicles appear to constitute substantial concerns for bicyclists.

Often-mentioned reasons not to bicycle are safety concerns. A large part of these safety concerns are most probably related to motor vehicles. *Traffic: unsafe or safe *contributed positively to the variance of the outcome variable when it was included in our analysis as a predictor. It took over the role of *congestion: all types of vehicles *as a significant predictor. This finding supports the influence of safety in stimulating bicycling behaviours cf. [13,25,26].

In line with these overall findings regarding road users and traffic concerns, bicyclists in general appear to have a preference for routes with a lower traffic volume and speed limits [27]. In a study of perceptions of motivators and deterrents of bicycling [14], 'streets with a lot of car, bus and truck traffic, vehicles driving faster than 50 km/h, risk of injury from car-bike collisions, and risk from motorists who do not know how to drive safely near bicycles' were among the top deterrents. 'Routes away from traffic noise and air pollution' was, on the other hand, ranked as the strongest motivator. In contrast, air pollution did not seem to be a reason for bicyclists to take a detour [24], and objectively measured traffic speed and volume were not related to the likelihood of bicycling [23]. These intuitively contradictory findings could be due to a lack of variability in the measured characteristics or a stronger influence of other factors on the decision to cycle. Still, given the design of the present study, it is reasonable to conclude that motor traffic as well as non-safety issues constitute hindering factors for bicycle commuting.

### 'Directness' of the route

The final predictor that contributed to the variance of the outcome variable in the models was *course of the route*. It was measured by the question: 'To what extent do you feel that your cycle trip is made more difficult by the course of the route? For example, a course with many sharp turns, detours, changes in direction, side changeovers etc.' The inclusion of the item in the ACRES evolved from the theories of space syntax see [8], which state that the configuration of the street network in and of itself is a strong movement generator in relation to walking. The fewer the number of direction changes that the street network requires a person to make to reach a certain destination, the more the street configuration is believed to stimulate movement [28]. *Course of the route *could be interpreted as the 'directness' of the route, which could be related to connectivity. Moreover, in a broader sense, *course of the route *could relate to street connectivity. Greater street connectivity has shown an association with higher levels of physical activity for a review of reviews, see [15]. Street connectivity is also one of the factors that constitute walkability cf. [29,30]. Interestingly, connectivity has, as part of walkability attributes of the neighbourhood environment, recently been associated with transport bicycling [30]. If we interpret *course of the route *as the 'directness' of the route, related to connectivity, our finding is in accord with previous research.

### Bicycle-related infrastructure and bicycle paths

In general, bicyclists seem to prefer bicycle-related infrastructure, such as bicycle paths or lanes, which separates them from motor traffic. The preferences are most likely related to safety issues arising from contact with other road users, mainly motor vehicle drivers. For example, findings from a stated preference study demonstrated that bicycle commuters in general preferred routes with low volumes of motor traffic and routes separated from motor traffic [31]. Parked cars and car parking facilities can further affect the accessibility as well as the safety of the bicyclist. It seems that bicyclist commuters avoid routes where parking is permitted [31] and that bicyclists prefer no parking along the route [27].

In a survey of the Municipality of Stockholm, bicycle paths were referred to as an issue influencing the willingness to bicycle more [32]. It was therefore somewhat unexpected that the item *bicycle paths/lanes/roads *did not contribute to the variance of the outcome variable in our study. We believe that the reason for this could be that the question in this matter includes a mix of bicycle-related infrastructures. It was measured with the question: 'About how large a part of your route consists of cycle paths/cycle lanes/cycle roads separated from motor-car traffic?' Thus, we ask for a total level of a mix of bicycle-related infrastructures. In Greater Stockholm a substantial part of this mix consists of cycle lanes, which is not the preferred cycling facility among bicycle commuters, who prefer bicycle paths instead [33]. Thus, the way we have posed the question might hide a potential positive effect of bicycle paths *per se *on the outcome variable.

### The continuity of the movement of the bicycle trip and red lights

The continuity of the movement of the bicycle trip is another aspect associated with bicycle-related infrastructure which could influence bicycling. The direction of the association is ambiguous, however, because traffic controls or traffic calming could probably influence bicyclists both negatively and positively. Negatively in terms of interrupting the bicycling flow, and positively in terms of convenience and safety cf. [25,34]. Our item *red lights *was measured by the question 'To what extent do you feel that your progress in traffic is worsened by the number of red lights during your trip to your place of work/study?', implying the negative aspect. Although *red lights *was not one of the predictors that contributed to the variance of the outcome variable, it showed a negative correlation with the outcome variable, possibly reflecting the negative aspect of traffic controls on the continuity of the movement of the bicycle trip.

### Hilliness

The hilliness of the route could have an impact on bicycling. A terrain with many slopes requires an extended effort of the bicyclist. Several studies have found a negative effect of slopes on bicycle use for an overview, see [25]. In contrast, there are some studies that have indicated contradictory results [23,27,31,35]. One possible explanation for the contradictory results is that the purpose of the trip for some bicyclists is, at least partly, to get exercise. Consequently, hilly terrains may therefore be preferred. In our study, the item *hilliness *showed a zero correlation with the outcome variable. A possible explanation is a lack of hills in the measured environment. The inner urban parts of Greater Stockholm are rather flat, and the infrequent existing hills most often constitute gentle slopes.

### Generalizability to other groups

To what extent might these results possibly be generalized to other groups? The work with the ACRES is in a relatively early stage at present. Studies are therefore also desirable regarding active transports with other purposes, different route environments and different samples. In this respect, we believe, however, that the results are valid for at least potential new bicyclists, i.e. current non-cyclists who are willing to start cycling, as well as for occasional - regular bicyclists in another metropolitan setting. This belief stems from a study of bicyclists' preferences based on viewing images of a great variety of different types of existing transport infrastructure in Vancouver, Canada [36]. The rank orders of preferences of the different route types, expressed as the likelihood to make use of them, were similar between different groups of current bicyclists and potential bicyclists. We have had the opportunity to scrutinize the images in the questionnaire used in their study and have compared them with our own findings. And, indeed, the extent to which our stimulating and hindering factors (the items *course of the route *and *ugly or beautiful *not being judged) are present in the images, or can easily be evoked by associations with ingredients in the images, mirror the ratings by the respondents in Vancouver. Interestingly, however, current bicyclists generally appeared to be more likely to make use of all different route types than the potentially new ones in their study. This opens up different interpretations. The potential and current bicyclists may represent subgroups within the population with different perceptions and/or interpretations of route environments. Another possibility is that one changes one's perception and/or interpretation of route environments with usage of them. Indeed, to further our understanding of these matters is an important target for future research.

### Limitations and strengths

This study has some possible limitations. First, it was solely based on perceptions of the route environment. There are a number of potential biases to consider when working with self-report questionnaires, cf. [37]. Both objective aspects and people's perceptions of the environment can be assessed for a review, see [38]. Naturally, more objective measurements may provide additional information. Nevertheless, it is important to study perceptions of the environments since they are likely to influence people's physical activity behaviours, cf. [4]. For example, if people think that the traffic environment is unsafe, their perceptions could result in a non-active commuting behaviour even though the environment is safe in some objective sense. Normally, active commuting is a repetitive behaviour along a specific route. Consequently, active commuters will most likely become very familiar with their particular route environments, and therefore their perceptions of the route environments can be considered relevant and may possibly further our understanding of the relationship between active commuting and the route environments. Furthermore, studies have shown poor agreement between objective and perceived measures of environments [39-42].

Second, the representativity of the study sample could, in principle, be limited. In general, active commuters represent a small proportion of the population in larger cities. Consequently, it is difficult to use population-based random samples when the aim is to study this group. In this study we used advertisement recruitment as the sampling method. Since we were concerned about the representativity, we have compared this method with street recruitment as a sampling method in a previous study [7]. The street-recruitment method was considered to represent the population of active commuters with greater certainty than the advertisement-recruitment method. Overall, the results indicated good correspondence between the advertisement- and street-recruited participants' ratings of the route environments. Although not tested for differences, the descriptive characteristics of both participant groups yielded a very homogeneous picture. Altogether, this strengthens the use of the advertisement-recruited sample.

Third, the statistical approach used in this study might be a limitation. As mentioned before, our work is in a relatively early and exploratory stage and therefore a simultaneous multiple regression analysis was regarded as appropriate. We felt that we did not, at this stage, have a sufficient amount of theoretical explanations to use a hierarchical approach. Hierarchal multiple regression analyses, as well as path analyses, possibly based on factor analyses, are desirable future approaches. These approaches, as well as analyses of interactions of variables, may possibly further the general state of knowledge and understanding of mediators, moderators and confounders in relation to the possible associations between bicycle commuting and route environments.

Apart from the above-mentioned possible limitations, this study has several strengths. Some of them are due to our research approach, already discussed at the beginning of this Discussion. One substantial strength is that we have used the ACRES. It has, compared to other questionnaires developed to assess the possible relationship between physical activity and the environment, e.g. [43,44], more points in the response scales than normally recommended, cf. [37]. In order to be able to carry out correlation studies between predictor and outcome variables, the ACRES has 15-point response scales. In addition, these scales have, in principle, the potential to capture changes and associations of rather fine distinctions. The use of the scale has been strengthened by our previous validity and reliability assessments [7,8]. Furthermore, most other questionnaires in the research field define the measured environmental area as the local neighbourhood, e.g. [43,44]. Active commuting, however, often involves an extended area. We have therefore used the ACRES, which considers the whole commuting route environment and has complete spatial matching between the environment and the physical activity variable [8]. An additional strength is that our participants were bicycle commuters, and thus experts on their own route environments. The research approach we used, i.e. studying a specific behaviour and the environment within which the behaviours is performed using specific measures, is emphasized and recommended by Giles-Corti and colleagues [6].

### Future studies

The work with the ACRES is, at present, in a relatively early stage and we have only performed exploratory analyses based on the inner urban area of one city and on one sample of people. A future approach is to study the suburban parts of Greater Stockholm, which, interestingly, as previously mentioned, have a clearly different commuting route environment profile, indicating higher bikeability, compared to the inner urban areas [7]. Furthermore, including perceived exertion and route distance as factors that might affect the perception of environments represents interesting perspectives. We also believe that it is important to sort out the different dimensions included in the concept of aesthetics. Studies are also desirable regarding active transports with other specific purposes, different route environments and different samples, including people with different experiences of active transport. Indeed, much is to be learned about the relationship between physical activity and the environment.

## Conclusions

In conclusion, the main results indicate that beautiful, green and safe route environments seem to be, independently of each other, stimulating factors for bicycle commuting in inner urban areas of a metropolitan setting. On the other hand, exhaust fumes, traffic congestion and low 'directness' of the route, seem to be hindrances to bicycle commuting. Irrespective of whether these factors have the potential to change behaviours, they affect the well-being of bicyclists when commuting in their route environments. In our mind, the results constitute a sound basis for urban planners to consider when aiming at enhancing these dimensions of the route environments for bicycle commuters.

## Competing interests

The authors declare that they have no competing interests.

## Authors' contributions

PS and LW designed the study. PS was involved in the data acquisition. LW checked the data from the PACS Q2 for accuracy, performed the statistical analyses and drafted the first version of the manuscript. PS drafted the manuscript and supervised LW as part of her PhD training. Both authors read and approved the final manuscript.

## Pre-publication history

The pre-publication history for this paper can be accessed here:

http://www.biomedcentral.com/1471-2458/12/168/prepub
